# RETRACTED: Esmaeilzadeh et al. Identify Biomarkers and Design Effective Multi-Target Drugs in Ovarian Cancer: Hit Network-Target Sets Model Optimizing. *Biology* 2022, *11*, 1851

**DOI:** 10.3390/biology13020086

**Published:** 2024-01-30

**Authors:** Biology Editorial Office

**Affiliations:** MDPI, St. Alban-Anlage 66, 4052 Basel, Switzerland; biology@mdpi.com

The journal retracts the article, (Identify Biomarkers and Design Effective Multi-Target Drugs in Ovarian Cancer: Hit Network-Target Sets Model Optimizing. *Biology* 2022, *11*, 1851) [1] cited above.

Following publication, concerns were brought to the attention of the publisher relating to the authenticity of the authorship of this article [1].

Adhering to our complaints procedure, an investigation was conducted by the Editorial Office and Editorial Board, who were unable to verify the identity, contribution, or affiliations of a number of the authors listed on this manuscript. MDPI follows the International Committee of Medical Journal Editors (ICMJE) guidelines when it comes to acknowledging authorship contribution (https://www.mdpi.com/ethics#_bookmark3, accessed on 16 January 2024). In this case, neither the authors nor the authors’ institutions could satisfactorily establish the contribution that they made to this article, nor could the origins of this study be verified. As a consequence, the Editorial Board has lost confidence in the integrity of the findings and has decided to retract this study as per MDPI’s retraction policy (https://www.mdpi.com/ethics#_bookmark30, accessed on 16 January 2024). 

This retraction was approved by the Editor-in-Chief of the journal *Biology.*

The authors did not provide a comment on this retraction decision.

## References

[1] Esmaeilzadeh A.A., Kashian M., Salman H.M., Alsaffar M.F., Jaber M.M., Soltani S., Ilhan A., Bahrami A. (2022). RETRACTED: Identify Biomarkers and Design Effective Multi-Target Drugs in Ovarian Cancer: Hit Network-Target Sets Model Optimizing. Biology.

[2] Biology Editorial Office (2024). Correction: Esmaeilzadeh et al. RETRACTED: Identify Biomarkers and Design Effective Multi-Target Drugs in Ovarian Cancer: Hit Network-Target Sets Model Optimizing. *Biology* 2022, *11*, 1851. Biology.

